# Experimental Investigation on the Improvement of Dredged Sludge Using Air–Booster Vacuum Preloading with Polyacrylamide Addition

**DOI:** 10.3390/ma18092065

**Published:** 2025-04-30

**Authors:** Heng Zhang, Lingfeng Guo, Chongzhi Tu

**Affiliations:** 1 School of Civil Engineering and Architecture, Wuyi University, Jiangmen 529000, China; zhanghengxyy@163.com; 2 School of Civil Engineering and Transportation, South China University of Technology, Guangzhou 510641, China; glfcoms@outlook.com

**Keywords:** soft soil, air–booster vacuum preloading, polyacrylamide, water discharge, soil particle morphology

## Abstract

Reducing the water content in soft soil is crucial for improving its load-bearing capacity. However, traditional vacuum preloading demonstrates limited effectiveness for dredged sludge due to its high water content and low permeability, resulting in inadequate consolidation and long treatment durations. To address these limitations, this study proposes a new improvement approach that combines pressurized air injection with a polyacrylamide (PAM) addition to enhance vacuum consolidation. Experimental results demonstrated that cationic polyacrylamide (CPAM) exhibited superior performance in improving water discharge efficiency, which promoted the aggregation of fine soil particles and reduced the clogging of drainage channels through adsorption bridging. The incorporation of pressurized air injection further enhanced consolidation efficiency by increasing hydraulic gradients and inducing micro-fractures in soil, thereby improving soil permeability and vacuum pressure transmission. However, excessive CPAM addition or high-pressure air injection was found to compromise the effectiveness of the vacuum preloading treatment due to drainage channel clogging and extensive soil fracturing. The appropriate consolidation performance was achieved with a 0.075% CPAM addition and 20 kPa air pressure injection, demonstrating a 24.5% increase in water discharge mass and a 30.9% improvement in soil shear strength compared to traditional methods. Microstructural analysis revealed a more compacted soil matrix with reduced porosity and enhanced interparticle interactions. These findings provide valuable insights for improving the treatment efficiency of dredged sludge in coastal regions, particularly in the Nansha District of Guangzhou.

## 1. Introduction

The effective reinforcement of soft soil foundations has emerged as a critical challenge in coastal urbanization development [1,2,3], particularly in China’s Pearl River Delta region where the Guangdong–Hong Kong–Macao Greater Bay Area construction project has been rapidly progressing since 2019. The geological conditions in this region are characterized by deeply buried soft soil layers resulting from long-term alluviation and sedimentation processes, with the local thickness exceeding 50 m in some areas. Soft soil typically demonstrates unfavorable engineering properties, including high water content, low permeability, and low bearing capacity, posing substantial challenges for geotechnical applications such as foundation engineering and slope stabilization [4,5]. Currently, vacuum preloading has been widely adopted as an effective technique for soft soil consolidation [6,7]. This method increases the hydraulic gradient within the soil matrix by generating negative pressure, thereby accelerating pore water discharge. However, in engineering practice, the extremely low permeability of soft soil causes a significant decay in vacuum degree with increasing depth and width, thus limiting the range of soil consolidation. Furthermore, the migration and accumulation of fine soil particles under vacuum gradient readily cause the blockage of drainage channels, leading to insufficient levels of soil consolidation when using vacuum preloading [8,9,10]. Consequently, developing a rapid and efficient approach for consolidating soft soil is urgently necessary.

In the process of vacuum preloading, the driving force for pore water migration originates from the pressure gradient in the soil matrix, and therefore, a higher pressure gradient generally results in greater water discharge. Based on this principle, many scholars have introduced pressurized air into soil to increase the pressure gradient, thereby promoting drainage and consolidation. This technique is referred to as air–booster vacuum preloading (AVP) and has attracted significant interest. For instance, Cai et al. [11] demonstrated the effectiveness of AVP by utilizing prefabricated vertical drains (PVDs) as inflow channels for pressurized air. This approach significantly improved the physical and mechanical properties of soft soil, such as permeability and undrained shear strength. Similarly, Lei et al. [12] compared three modified vacuum preloading techniques and found that AVP outperformed other methods in consolidating ultra-soft soil, particularly in terms of the drainage efficiency and consolidation rate. Throughout the current research, the superiority of AVP in consolidating soft soil has been demonstrated. First, the injection of pressurized air increases the pressure difference within the soil, promoting the hydraulic gradient to accelerate water discharge. Second, the pressurized air creates micro-fractures in the soil matrix, leading to improved permeability and more effective vacuum pressure transmission. Third, the pressurized air exerts a pushing force on fine particles blocking the filter openings, reducing drainage channel clogging [13]. Through the above mechanisms, larger amounts of water were discharged, thereby accelerating the soil consolidation. However, AVP can only remove free water from soil pores and is unable to extract weakly bound water, which significantly influences the engineering properties of soft soils [14]. This limitation reduces the effectiveness of AVP in drainage and consolidation, particularly for fine-grained soils. Moreover, the success of AVP largely depends on the pressure of the pressurized air. Both excessively high and low pressures can compromise the effectiveness of this method. In current AVP research, air pressure is typically determined empirically, which may not always obtain optimal results [11,15].

In recent years, polymers have been widely used in various aspects of our daily lives, and their application in geotechnical engineering has attracted increasing attention, particularly as additives for soil stabilization [16,17,18]. Soil is a porous structure whose strength is primarily derived from the skeleton composed of soil particle agglomerates. Therefore, the mechanical behavior of soil under external forces can be significantly enhanced by strengthening the physical and mechanical properties of the soil particles. From this perspective, the adsorption of polymers onto clay mineral surfaces changes particle properties. This change in interparticle forces provides a viable method for soft soil improvement. Among the various polymers used for this purpose, polyacrylamide (PAM) has emerged as a cost-effective and versatile option, widely used in industrial applications [19]. As a high-molecular-weight synthetic polymer, PAM significantly improves the mechanical and physical properties of soil particles through neutralization and adsorption, inducing a fabric alteration process [20]. This process causes small soil particles to aggregate into larger clusters via PAM-induced flocculation, thereby improving soil permeability and enhancing soil consolidation efficiency.

To address the poor consolidation performance and long treatment duration of vacuum preloading in treating dredged sludge, this study proposes a synergistic method that combines air–booster vacuum preloading (AVP) and polyacrylamide (PAM) to improve the consolidation efficiency. To validate this approach, drainage tests were conducted to determine the appropriate types and dosage of PAM, and model tests were designed to assess the combined effects of AVP and PAM on the geotechnical properties of dredged sludge. Water discharge, vacuum pressure, and surface settlement were monitored during vacuum preloading. The vane shear strength, water content, and microstructure of the treated soil were evaluated.

## 2. Materials and Methods

### 2.1. Materials

**Figure 1:**
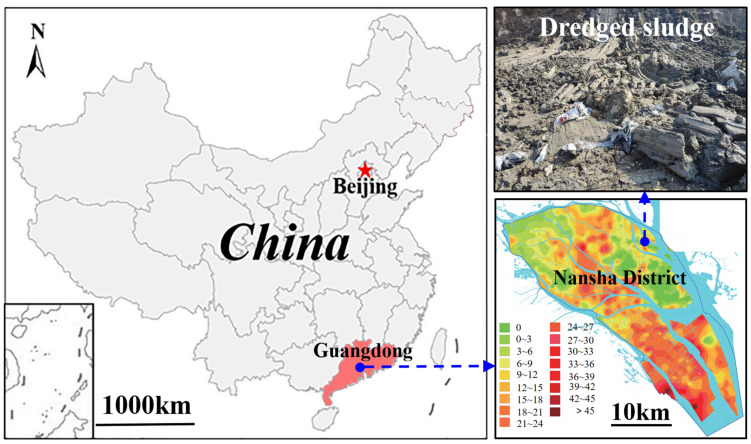
Geographic location of the sampling site.

**Figure 2:**
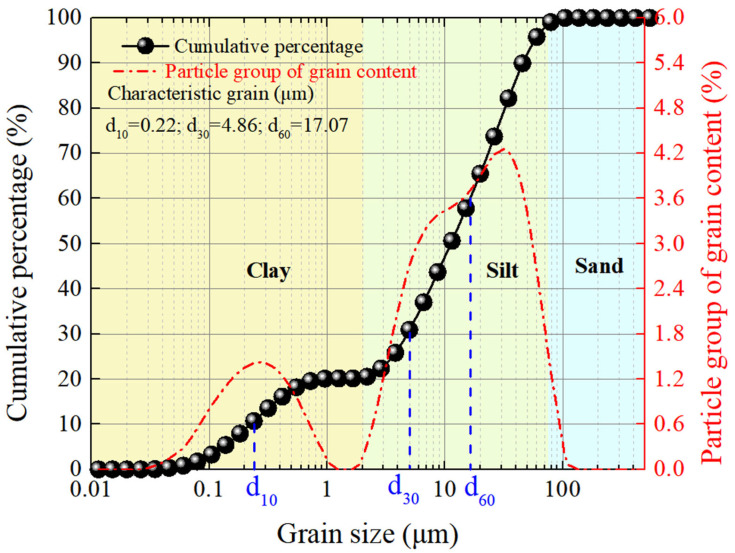
Grain size distribution of the studied soil.

The dredged sludge used in this experimental study was obtained from Nansha District (22°50′47″ N, 113°32′35″ E) of Guangzhou, China, an area characterized by extensive soft or ultra-soft soil deposits, as shown in Figure 1. Prior to laboratory testing, the natural soil was immersed in water to remove the organic impurities and oversized stones. Subsequently, the sludge was dehydrated in an oven and pulverized into powder. A calculated quantity of soil powder was thoroughly mixed with water in a stirrer to reach the predetermined water content [1,10,21]. The relevant physical properties of the reconstituted soil are summarized in Table 1. The liquid limit and plastic limit, measured using the Casagrande method and thread rolling, were 42.6% and 20.2% [22], respectively, with corresponding plasticity and liquidity indices of 22.4 and 2.26. Other index properties of the soil were also measured, such as the density (1.62 g/cm^3^), specific gravity (2.71), void ratio (1.92), and saturation (100%). The initial water content was 70.8%, 1.66 times the liquid limit; thus, the natural soil displayed a flow state with virtually no shear strength. The particle size distribution was determined using a laser particle size analyzer (Mastersizer 2000, Malvern Panalytical Ltd., Worcestershire, UK). As plotted in Figure 2, the proportions of sand (d > 75 μm), silt (2 μm < d < 75 μm), and clay (d < 2 μm) accounted for 1.5%, 77.9%, and 20.6% of the total soil mass, respectively, indicating that the soil was predominantly composed of fine particles. Hence, many extremely fine particles passed through the void pores driven by the vacuum pressure gradient, accumulating near the filter membrane surface and causing the blockage of drainage channels [5]. Based on the mentioned physical parameters, the soil was classified as low-plasticity clay (CL) according to the Unified Soil Classification System [23]. Additionally, three types of polyacrylamides (PAMs; molecular weight: 12 million), i.e., anionic (APAM), cationic (CPAM), and nonionic (NPAM), were purchased from Sinopharm Chemical Reagent Co., Ltd. (Shanghai, China) and used without further purification. The PAM was first dissolved in deionized water to prepare homogeneous solutions, which were then added to the prepared sludge and thoroughly mixed to obtain soil samples with the target PAM additions of 0.025%, 0.05%, 0.075%, and 0.1% by dry soil mass.

### 2.2. Air–Booster Vacuum Preloading System

**Figure 3:**
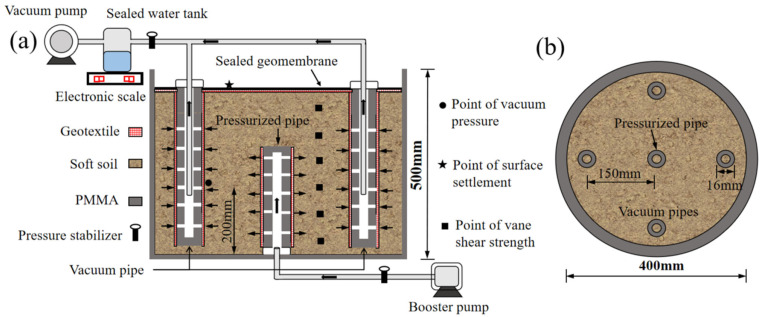
Schematic of the air–booster vacuum system. (**a**) Schematic of the model test equipment. (**b**) The cross-section profile of the model barrel.

The air–booster vacuum system mainly consisted of a model barrel, a booster pump, a vacuum pump, pressurized and vacuum pipes, a sealed water tank, transducers, and a data logger. The model barrel was made of a polymethyl methacrylate (PMMA) cylinder with a size of 400 mm in diameter and 500 mm in height and perforated with a 10 mm diameter hole at the bottom center. The utilized booster pump could control output pressure loads via a governor with an accuracy of ±0.5 kPa, and the vacuum pump could provide vacuum pressure loads over −85 kPa. Both the pressurized and vacuum pipes were made of PMMA tubes with an inner diameter of 12 mm, uniformly perforated with 1 mm diameter holes on the surface and wrapped with geotextile to enhance their discharge capacity and bending resistance. The pressurized pipe was positioned vertically in the barrel’s center to a 200 mm depth and connected directly to the booster pump through the bottom. Four vacuum pipes were evenly arranged around the central pressurized pipe at a spacing of 150 mm and connected to the sealed water tank with rubber tubes to collect discharged water. Transducers were buried at the desired positions during the preparation of the soil specimen by layering and subsequently connected to the data logger. A geomembrane and geotextile were used to cover the top surface of the sludge and create an airtight condition, respectively. The schematic of the air–booster vacuum system is shown in Figure 3.

### 2.3. Test Procedures

**Figure 4:**
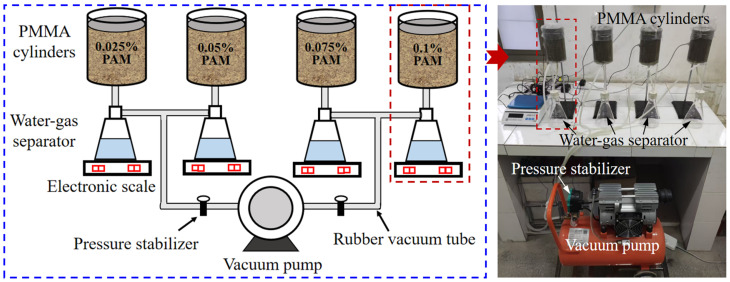
Schematic of the drainage test setup.

The experimental procedures in this study consisted of a drainage test and a model test. The drainage test was initially performed to identify the appropriate type and dosage of the PAM additive for the subsequent model test, which significantly reduced the consumption of time and materials. The drainage test setup included a vacuum pump, water–gas separators, 5 mm thick PMMA cylinders (80 mm in inner diameter and 160 mm in height), rubber vacuum tubes, and a geomembrane, as shown in Figure 4. Before applying the vacuum loading, three types of PAM solutions were, respectively, added to the prepared sludge and thoroughly agitated to obtain sludge with PAM additions of 0.025%, 0.05%, 0.075%, and 0.1% by dry soil mass. A permeable rigid pad wrapped with a geotextile was placed at the bottom of the cylinder to prevent the clogging of the drain hole. The sludge containing PAM, with a water content of 71.0%, was poured into the PMMA cylinder in layers and sealed with a geomembrane on the surface for 12 h to achieve a more uniform moisture and PAM distribution. Subsequently, a vacuum pressure of −85 kPa was applied through the bottom of the PMMA cylinder. The discharged water was collected into the water–gas separator and periodically measured using an electronic scale.

To ensure the reliability of the experimental data, the drainage test was repeated twice under identical conditions. By analyzing the water discharge in the drainage test, the appropriate additive and dosage for the following model test were determined. The resulting sludge was poured into the model barrel in layers and sealed with a geotextile and geomembrane on the surface for 24 h to reach a more uniform state. A vacuum pressure of −85 kPa was continuously applied to the soil specimens from the vacuum pump. Subsequently, pressurized air at 0 kPa, 20 kPa, and 40 kPa was activated when the water discharge rate dropped below the threshold of 50 g/h and was maintained for 2 h while the vacuum pump was operating. The model test was terminated when the duration reached 160 h, according to preliminary research. During the testing process, the mass of discharged water, vacuum pressure, and surface settlement were recorded periodically. The undrained shear strength of the treated soil was measured using an SZB-2.0 portable vane shear tester (Yinganyang Instrument Co., Ltd., Changzhou, China). The vane was first inserted into the soil to the target depth, then the handle was slowly rotated until shear failure occurred. The maximum torque was recorded to calculate the undrained shear strength. At each depth, the measurement was repeated three times, and the average value was taken as the final shear strength of the treated soil. Additionally, the soil microstructure was characterized using scanning electron microscopy (SEM, ZEISS SIGMA 500, Oberkochen, Germany). Prior to imaging, the soil specimens were sputter-coated with a gold layer to ensure electrical conductivity. All observations were conducted under high-vacuum conditions (≤5 × 10^−6^ mbar) with working distances maintained between 7 and 8 mm at an accelerating voltage of 5 kV. The water content was determined using the oven-drying method.

## 3. Results and Discussion

### 3.1. Discharged Water in Drainage Test

**Figure 5:**
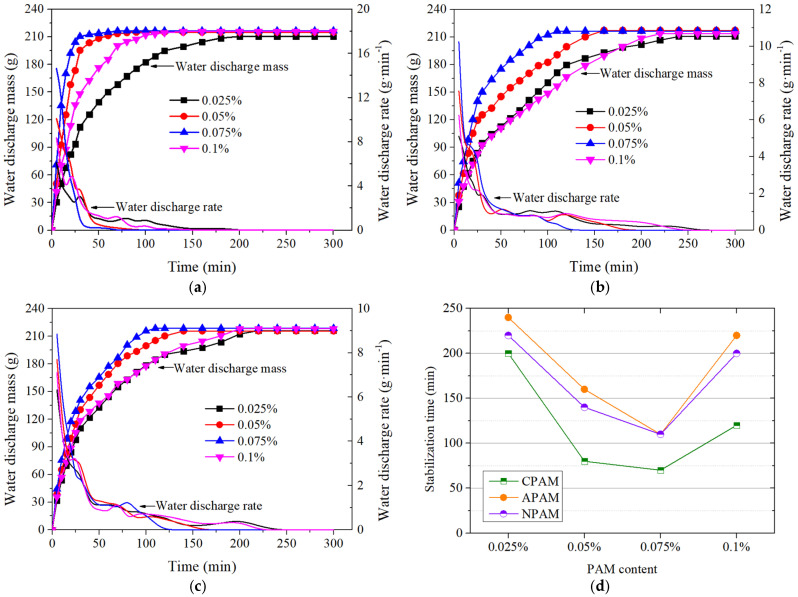
Water discharge characteristics with varying PAM contents. (**a**) Water discharge with CPAM addition. (**b**) Water discharge with APAM addition. (**c**) Water discharge with NPAM addition. (**d**) Stabilization time with different PAM contents.

Figure 5 shows the water discharge characteristics with varying PAM contents. Significant differences were observed in the water discharge efficiency. The time required to reach stability for all groups first decreased and then increased with the increase in PAM content. The minimum stabilization time of 70 min was achieved with a 0.075% CPAM addition, representing a 36.4% reduction compared to APAM and NPAM (110 min). However, the ultimate discharged water was nearly identical (approximately 210 ± 5 g), primarily due to the finite sludge volume. In addition, the water discharge rates for all groups gradually decreased and fluctuated within a certain range. The average water discharge rates during the early stage ranged from 3.6 to 9.6 g/min. Notably, the group with a 0.075% CPAM addition exhibited the highest rate of 9.6 g/min, which was nearly 60% higher than those of the APAM or NPAM groups. These results highlight the beneficial effect and appropriate dosage of the CPAM addition.

**Figure 6:**
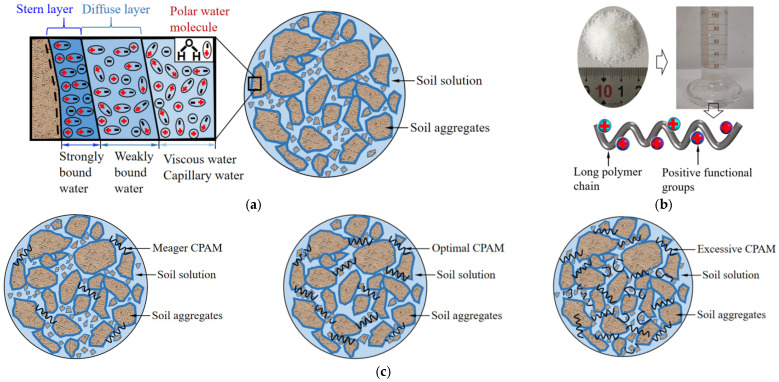
Schematic diagram of the soil particles with CPAM addition. (**a**) The initial state of soil particles. (**b**) Schematic diagram of CPAM additive. (**c**) The states of soil particles with different CPAM additions.

The drainage results can be primarily attributed to the following reasons. (1) Soil particle surfaces are negatively charged due to isomorphous substitution and ion exchange [24], which attracts polar water molecules to form double electric layers (stern layer and diffuse layer). The diffuse layer degrades soil permeability, shear strength, and compressibility, making sludge consolidation more difficult [25]. (2) CPAM contains numerous positively charged functional groups (quaternary ammonium and tertiary amine) that neutralize the negative charges on soil particles [26]. This neutralization reduces interparticle repulsion forces and decreases the diffuse layer thickness, thereby promoting soil particle aggregation [27]. Furthermore, CPAM’s long molecular chains capture soil aggregates through electrostatic attraction, forming a flocculated network structure. Consequently, CPAM demonstrates superior flocculation efficiency compared to NPAM and APAM. (3) When the CPAM content exceeds 0.075% and reaches 0.1%, the excess positive charge once again surrounds the soil particles [5]. This results in the charge reversion of fine soil particles and greatly diminishes their aggregation due to electrostatic repulsion [28,29]. Moreover, excessive CPAM content increases the fluid viscosity, impeding the movement of water through soil pores [12,30]. Conversely, when the CPAM content is insufficient, it cannot prevent additional soil particles from accumulating near the filter membrane. Schematic diagrams illustrating these mechanisms are presented in Figure 6. Under the synergistic effects of charge neutralization and particle aggregation, a significant improvement in the consolidation of dredged sludge was achieved with a CPAM addition of 0.075% by mass.

Based on the above analyses, five groups of vacuum preloading model tests were carried out simultaneously. Among these, three groups were treated with a 0.075% CPAM addition and pressurized air at 0, 20, and 40 kPa, respectively. The remaining two groups served as control tests, treated by traditional vacuum preloading with and without CPAM additions. For simplicity, the model tests were referred to as TVP, CVP, AVP-0, AVP-20, and AVP-40, corresponding to the different consolidation methods. The experimental conditions for all soil samples are summarized in Table 2.

### 3.2. Discharged Water in Model Test

**Figure 7:**
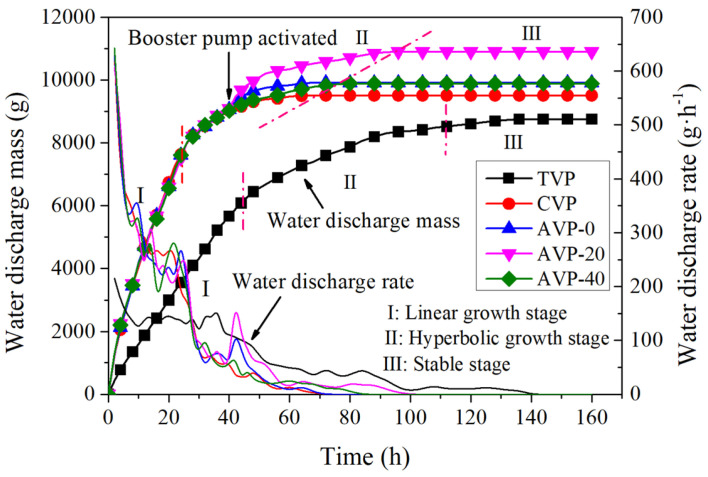
Temporal evolution of water discharge in model tests.

Water discharge plays a critical role in evaluating the effectiveness and efficiency of vacuum preloading. Figure 7 depicts the temporal variation in water discharge mass during vacuum preloading with the CPAM addition and pressurized air application. It shows that the cumulative mass of discharged water can be divided into three distinct stages: a linear growth stage, a transition stage, and a stable stage. In the first stage, the water discharge of CVP increased sharply, with a significantly higher growth rate (7614 g in 24 h) compared to that of TVP (6276 g in 46 h), indicating that CPAM dramatically accelerated the water discharge process. Prior to booster pump activation, the trends of water discharge increase in pressurized groups were nearly identical to that of CVP. As the water content continued to decrease, the van der Waals forces between water molecules and soil particles increased, making further water discharge more energy intensive. Consequently, the water discharge curve exhibited a downward curvature, accompanied by a decline in the drainage rate. When the water discharge rate dropped below 50 g/h, the booster pump was activated. The introduction of pressurized air created an additional positive pressure difference between the pressurized pipe and the vacuum pipe, increasing the hydraulic gradient within the soil and leading to a further increase in the water discharge. As a result, the cumulative water discharge curves showed a secondary rise. It is worth noting that this additional increase in water discharge was closely associated with the application of pressurized air. Theoretically, higher pressurized air should result in a greater increase in water discharge. However, for AVP-40, only a rapid but limited increase in water discharge was observed. In contrast, AVP-0 and AVP-20 showed higher increases in water discharge during booster pump operation. The underlying mechanism can be explained as follows: high-pressure air penetrated the soil and flowed upward to the geomembrane, resulting in the direct discharge of pressurized air through the vacuum pipe. This was confirmed by the temporary bulging of the geomembrane during pressurization. Furthermore, the high-pressure air fractured the soil, with microcracks initiating and extending into macrocracks during pressurization [13]. This process created airflow channels between the pressurized pipe and the vacuum pipe, accelerating the dissipation of pressurized air and reducing its effectiveness in compressing the soil. The ultimate water discharge masses for TVP, CVP, AVP-0, AVP-20, and AVP-40 were 8759 g, 9514 g, 9917 g, 10,904 g, and 9894 g, respectively. The pressurized groups discharged more water compared to the traditional vacuum preloading group. Notably, the water discharge mass of AVP-20 was approximately 110% of AVP-0 and AVP-40. Figure 7 reveals the benefits of combining pressurized air injection and the CPAM addition for improving water discharge efficiency during vacuum preloading. The addition of CPAM reduced the fine soil particle content through adsorption bridging [31], effectively alleviating drainage path clogging caused by the migration and agglomeration of fine soil particles. Additionally, the application of appropriate pressurized air exerted a squeezing effect, increasing the hydraulic gradient and creating micro-fractures to improve soil permeability. Consequently, 24.5% larger amounts of water were discharged.

### 3.3. Vacuum Pressure

**Figure 8:**
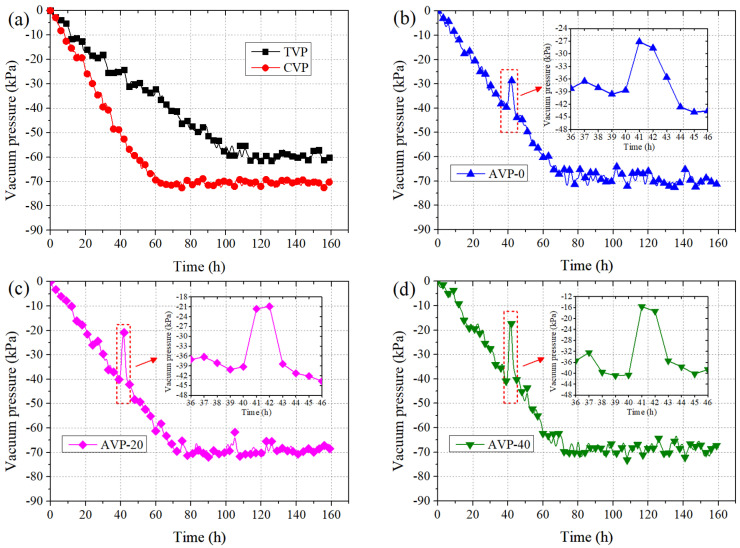
Variation in vacuum pressures vs. time. (**a**) TVP and CVP (**b**) AVP-0 (**c**) AVP-20 (**d**) AVP-40.

Vacuum pressure represents the difference between absolute pressure and atmospheric pressure. In this model test, a transducer was embedded into the soil specimen at a depth of 20 cm in each barrel. Figure 8 depicts the temporal variation in vacuum pressure near the vacuum pipe in five groups. It showed that the vacuum pressure of TVP increased slowly and eventually stabilized at around −60 kPa after 100 h. In contrast, for CVP, a much faster increase in vacuum pressure was presented, and the vacuum pressure increased almost linearly during the first 60 h. The ultimate vacuum pressure stabilized at around −71 kPa, indicating significantly higher efficiency in vacuum pressure transmission due to the improvement in soil permeability resulting from the CPAM addition. For the pressurized groups, the vacuum pressure curves were nearly identical to that of CVP before booster pump activation, after which the vacuum pressure fluctuated due to the pressurized air injection. These fluctuations were closely related to the pressure of pressurized air, as shown in Figure 8c,d. During booster pump activation, the vacuum pressures reduced by 12, 19, and 25 kPa for AVP-0, AVP-20, and AVP-40, respectively. This was due to the positive pressure produced by injecting pressurized air, offsetting the negative pressure from vacuum preloading, leading to a decrease in vacuum pressure. Notably, the vacuum pressure declines of AVP-0 and AVP-40 reached the smallest and the largest values, respectively.

### 3.4. Surface Settlement

**Figure 9:**
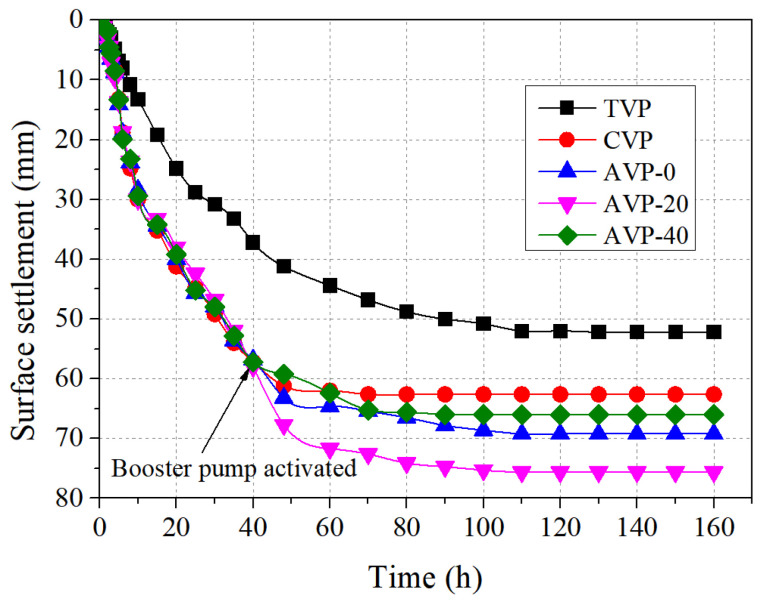
Evolution of soil settlement with time.

**Figure 10:**
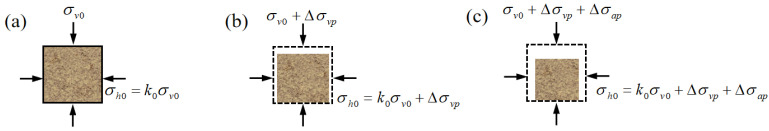
Stress states of the soil element subjected to vacuum pressure and booster pressure. (**a**) Initial stress state. (**b**) Stress state with vacuum preloading. (**c**) Stress state with air–booster vacuum preloading.

Figure 9 plots the curves of surface settlement over time for the five groups. The patterns in settlement are similar to those in cumulative water discharge. As shown, the settlement of CVP rapidly increased to 62.6 mm, which was 19.9% larger than that of TVP (52.2 mm). This improvement was attributed to the enhanced soil permeability resulting from the CPAM addition, leading to faster and better consolidation. Before the booster pump activation, the settlement curves of the pressurized groups largely overlapped with that of CVP. However, significant differences emerged during the pressurization process. For AVP-40, the high-pressure air induced the failure of efficient soil compression, resulting in a slightly smaller settlement compared to CVP in the early stage. This indicates that the high-pressure air negatively impacts soil consolidation. However, as the consolidation process advanced, a positive influence of pressurized air on soil consolidation occurred, and the surface settlement of AVP-40 surpassed that of CVP. In contrast, AVP-0 and AVP-20 exhibited a consistently positive impact of pressurized air on soil consolidation throughout the process. The ultimate settlements of AVP-0, AVP-20, and AVP-40 reached 69.2 mm, 75.6 mm, and 66.0 mm, respectively, representing 110, 121, and 105% times the settlement of CVP. These results indicate that pressurized air generally enhanced the consolidation of dredged sludge. This improvement can be primarily attributed to variations in the stress states of soil elements during testing, as illustrated in Figure 10. Initially, the soil element was in equilibrium under the combined action of vertical and horizontal stresses (*σ*_v0_ and *σ*_h0_). The application of vacuum preloading imposed an additional isotropic incremental stress (Δ*σ*_vp_) on the soil element, inducing consolidation accompanied by vertical settlement. Upon the activation of the booster pump, another isotropic incremental stress (Δ*σ*_ap_) was applied, further promoting soil consolidation and deformation. During pressurization, the booster airflow disturbed the soil particles without causing significant damage to the soil microstructure. Thus, the soil particles approached a new steady state with a reduced void ratio, consistent with the principle of “disturbances induce consolidation”.

### 3.5. Water Content

**Figure 11:**
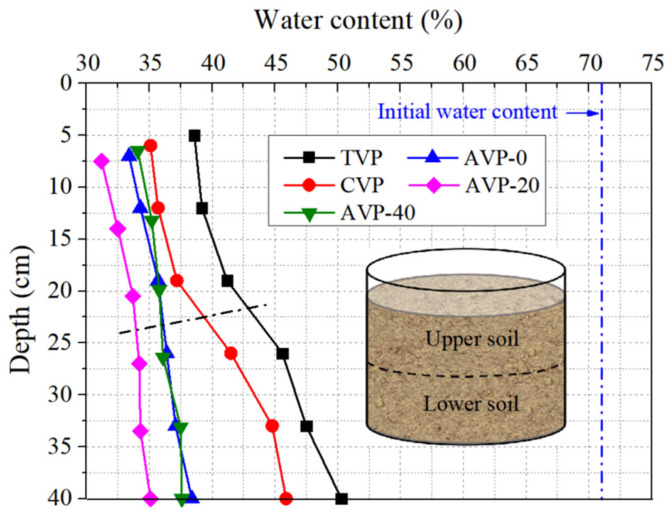
Changes in soil water content with depth after a 160 h treatment.

After the completion of vacuum preloading, soil samples were carefully extracted from six different depths to measure their water contents. Each measurement was repeated three times. Figure 11 illustrates the variation in water content with depth for the five experimental groups. Compared to their initial values, all water contents were significantly reduced. For instance, the average water content of the upper soil in TVP, CVP, AVP-0, AVP-20, and AVP-40 decreased to 39.6 ± 2.1%, 36.0 ± 2.5%, 34.5 ± 2.6%, 32.5 ± 2.1%, and 35.0 ± 2.3%, while the lower soil water content decreased to 47.8 ± 1.8%, 44.1 ± 2.4%, 37.3 ± 2.8%, 34.5 ± 2.4%, and 37.1 ± 2.0%, respectively. Compared to TVP, the water content of the upper and lower soil in CVP decreased by 3.6% and 3.7%, respectively, further demonstrating the beneficial effect of the CPAM addition. The differences in water content between the upper and lower soil were nearly 8.0% for TVP and CVP because of vacuum loss with increasing depth. In contrast, these differences were approximately 2.5% for the pressurized groups. Additionally, the soil water content in the pressurized groups was significantly lower than that in CVP at all depths, indicating that air–booster vacuum preloading achieved more effective and uniform soil consolidation. Notably, AVP-20 exhibited the lowest water content, with reductions of 7.1% and 3.5% in the upper soil and 13.3% and 9.6% in the lower soil compared to TVP and CVP, respectively. These variations in post-treatment soil water content were consistent with the water discharge measurements.

### 3.6. Shear Strength

**Figure 12:**
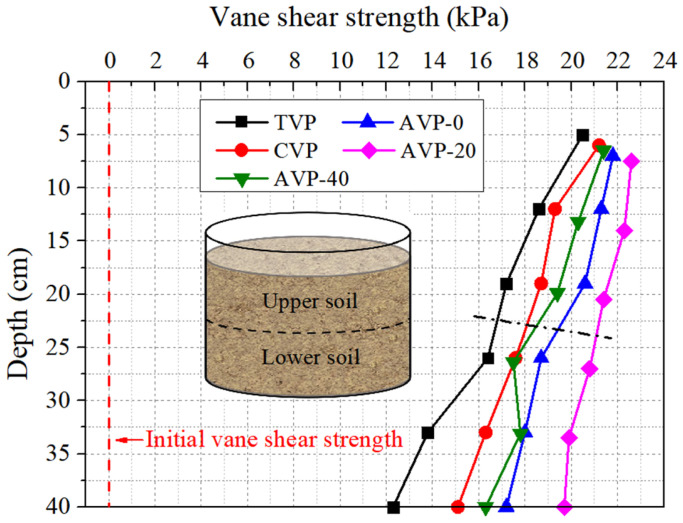
Changes in vane shear strength with depth after 160 h treatment.

Shear strength is a critical parameter for evaluating soil reinforcement. Figure 12 shows the results averaged based on three measurements. Significant enhancements were observed in the soil at different depths of five groups. The vane shear strengths of the upper and lower soil in CVP increased to 19.7 ± 2.8 and 16.3 ± 2.6 kPa, respectively, representing increases of 5.3% and 14.8% compared to those of TVP (18.7 ± 1.4 and 14.2 ± 2.0 kPa). This demonstrates the positive influence of CPAM addition on soil reinforcement. Moreover, the increments in vane shear strength for the pressurized groups were greater than those of CVP, reflecting the beneficial reinforcement effect of pressurized air. Notably, the soils from AVP-20 exhibited the highest vane shear strength at all depths, consistent with the trends observed in the water content results. Specifically, the vane shear strengths of the upper soil in AVP-0, AVP-20, and AVP-40 increased to 21.2 ± 3.0, 22.1 ± 1.9, and 20.4 ± 2.8 kPa, respectively, while the lower soil strengths increased to 18.0 ± 2.0, 20.1 ± 2.8, and 17.2 ± 2.3 kPa. The differences in vane shear strength between the surface soil and bottom soil were 8.2, 6.1, 4.6, 2.9, and 5.1 kPa for TVP, CVP, AVP-0, AVP-20, and AVP-40, respectively. A notable reduction in the vane shear strength difference was observed for the pressurized groups. The vertical differentiation of vane shear strength arises from depth-dependent water content variations, driven by vacuum loss and gravitational water redistribution [11]. Pressurized air injection mitigates these effects by enhancing vacuum transmission to lower soil, while CPAM improves permeability through flocculation, collectively reducing water content gradients and improving strength uniformity.

### 3.7. Soil Particle

**Figure 13:**
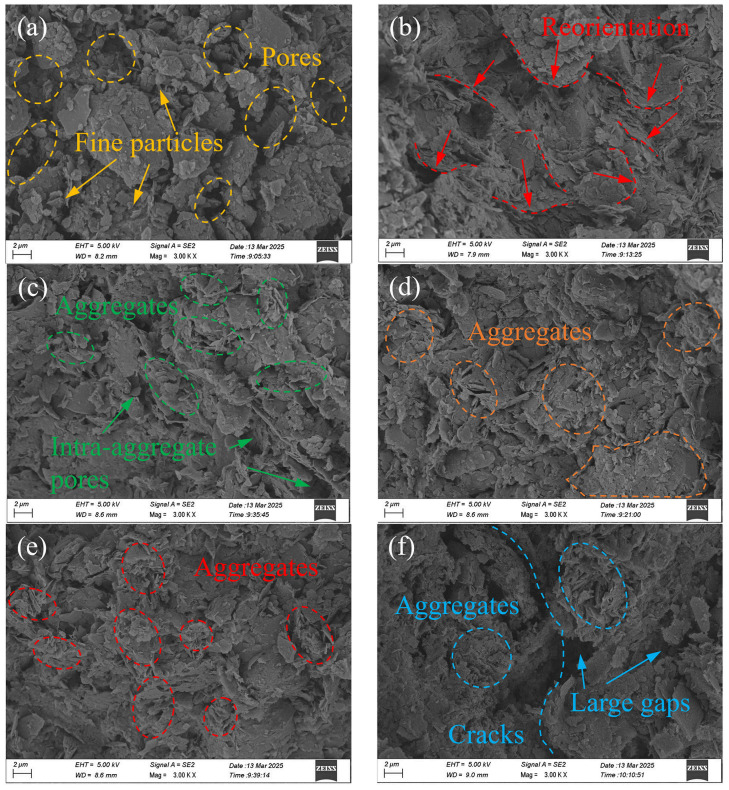
Representative micrographs of the soil specimens subjected to different consolidation methods: (**a**) natural soil; (**b**) TVP; (**c**) CVP; (**d**) AVP-0; (**e**) AVP-20; (**f**) AVP-40.

Furthermore, natural soil and post-treatment soil at a depth of 20 cm were selected for microscopic morphology analysis using SEM. Figure 13 presents a representation of multiple observations obtained from different samples, taken at 3000 magnification. As shown in Figure 13a, the natural soil exhibited a relatively loose and broken microstructure composed of numerous fine particles that were randomly distributed and rarely in contact with each other. Therefore, these fine particles were prone to migration under the influence of water flow (seepage) driven by a vacuum pressure gradient. Figure 13b shows the microscopic morphology of TVP. Compared to the natural soil, more platy faces of soil particles were observed, which aggregated in layers to form an anisotropic microstructure under vacuum pressure. This particle reorientation decreased the radial permeability of the soil to some extent, inducing degraded consolidation in the later stages of vacuum preloading [1]. In contrast, for CVP, as depicted in Figure 13c, the reorientation of platy particles was significantly reduced. Large soil particle aggregates formed under the combined action of vacuum compression and CPAM cementation. These aggregates moved as a whole, with very few fine particles migrating under the vacuum pressure gradient. Additionally, the interconnected intra-aggregate pores likely functioned as seepage channels, significantly improving soil permeability. Figure 13d–f show the microscopic morphology of AVP-0, AVP-20, and AVP-40, respectively. Although the soil particle morphology of AVP-0 was still dominated by a platy structure, a denser microstructure was achieved due to the incremental squeezing effect induced by pressurized air. The soil particles of AVP-20 exhibited no preferential orientation caused by vacuum pressure. Instead, the skeleton particles were uniform in size and closely bonded, as the booster airflow disrupted the particle reorientation induced by vacuum pressure, allowing the soil to reach a new equilibrium state with a reduced void ratio, as described in Figure 10. For AVP-40, obvious cracks were observed, and the soil microstructure was relatively loose, with large gaps between particles. This was primarily due to the high-pressure gradient penetrating the soil structure, creating airflow channels between the pressurized air and vacuum pressure. With the pressurizing time increasing, some of these airflow channels gradually connected to form cracks, leading to the failure of vacuum preloading. These microscopic observations were consistent with the macroscopic soil properties, confirming that the synergy of the air booster and CPAM improved soil consolidation under vacuum conditions.

## 4. Conclusions

This study conducted a series of laboratory experiments to evaluate the combined effect of air booster application and polyacrylamide (PAM) addition on improving dredged sludge. The drainage test was performed in advance to determine the appropriate type and dosage of the PAM additive for the model test. A comprehensive monitoring system was assembled to record the variations in water discharge, surface settlement, and vacuum pressure during the model test. The water content, vane shear strength, and microscopic morphology of post-treatment soil were tested. By comparing the physical and mechanical characteristics of soil samples, the following conclusions were drawn.

(1) Cationic polyacrylamide (CPAM) was confirmed as the appropriate additive for consolidating dredged sludge, as it not only accelerated the water discharge process but also promoted the aggregation of fine soil particles through neutralization and adsorption. In comparison with APAM or NPAM, the addition of 0.075% CPAM increased water discharge efficiency by nearly 36.4%, leading to the faster consolidation of dredged sludge in the drainage test.
(2) Air–booster vacuum preloading (AVP) consolidated dredged sludge more efficiently, as it provided an additional hydraulic gradient that accelerated water discharge. However, this positive effect was highly dependent on the pressurized air pressure. Excessive pressure caused soil fracturing and the formation of airflow channels, significantly reducing the effectiveness of air–booster vacuum preloading. Conversely, insufficient pressure failed to disrupt soil particle reorientation under vacuum pressure, limiting improvements in soil permeability.
(3) The combination of AVP and the CPAM addition demonstrated superior effectiveness in improving the physical and mechanical properties of dredged sludge. The pressurized groups obtained much higher efficiency in water discharge and vacuum pressure transmission than the comparison groups during testing. Compared with soil treated with traditional vacuum preloading, the soil shear strength of the pressurized groups increased by 19.2% to 60.2%, the ultimate water content decreased by 3.6% to 13.3%, and the soil’s microscopic morphology became more compact and uniform. Notably, the synergistic application of 20 kPa pressurized air and 0.075% CPAM was recommended based on the aforementioned test results.

The experimental results demonstrate that the synergistic application of AVP and CPAM significantly enhances the consolidation efficiency of dredged sludge, with the optimized parameters serving as guidelines for field applications in Nansha District, Guangzhou. These findings hold promise for coastal reclamation, where this method could greatly reduce treatment duration. Furthermore, the observed microstructural improvements (enhanced compactness and uniformity) suggest long-term stability for engineered foundations. To facilitate practical application, future work will focus on validating this approach across diverse soil types, including river sediments and industrial sludges.

## Figures and Tables

**Table 1:** Physical properties of the natural soil.

Initial Water Content	Density	Specific Gravity	Saturation	Void Ratio	Liquid Limit	Plastic Limit
*w*_0_/%	*ρ*/g·cm^−3^	*G*_s_	*S*_r_/%	*e_0_*	*w*_L_/%	*w*_P_/%
70.8	1.62	2.71	100	1.92	42.6	20.2

**Table 2:** Summary table of model test schemes.

Group ID	Consolidation Method	CPAM Dosage (by Dry Soil Mass)	Booster Pressure (kPa)	Pressurizing Time (h)
TVP	Vacuum preloading	/	/	/
CVP	Vacuum preloading	0.075%	/	/
AVP-0	Air–booster vacuum preloading	0.075%	0	2
AVP-20	Air–booster vacuum preloading	0.075%	20	2
AVP-40	Air–booster vacuum preloading	0.075%	40	2

## Data Availability

The original contributions presented in this study are included in the article. Further inquiries can be directed to the corresponding author.
